# Remarks on the Causes, Symptoms, and Treatment of Diarrhœa, as It Appears at Wetumpka, Alabama

**Published:** 1845-09

**Authors:** James C. Harris

**Affiliations:** Wetumpka, Alabama


Art. II.—*Remarks on the Causes, Symptoms, and Treatment of Diarrhœa, as it appears at Wetumpka, Ala.* By James C. Harris, M.D.

The towns of East and West Wetumpka, connected by a fine and apparently durable bridge, are situated upon both sides of the Coosa river, at the foot of the falls of the same name, and head of steamboat navigation, in latitude 32° 30′ and longitude west from Washington, 9°. These have been by legislative enactment erected into, and constitute our city, containing upwards of 2,000 inhabitants, several schools, four churches, and the State Prison, together with immense water facilities for the propulsion of all kinds of machinery.

Thus situated, with many internal evidences of her own greatness, in the untiring zeal and enterprise of her citizens, surrounded upon all sides by an intelligent, and upon the west and south a dense and wealthy population, she holds out to the merchant, artizan, and capitalist, for a permanent location in their different avocations, a fair prospect of pecuniary reward.

The site of the western town is a level sandy plain, cut and interspersed with an occasional ravine and lagoon, terminating rather abruptly at the river-bank in a high bluff; not so, however, with the eastern; here a greater portion of the town is so completely hemmed in with a range of high hills extending its whole length and rising several hundred feet above the level of the river, that at many points there was scarcely space enough between their base and the water's edge, for a broad street and the erection of the necessary business buildings.

These hills sloping back with gentle acclivities, and terminating in level tops, afford most desirable sites for the erection of private residences, and from one of which to the admirer of the works of nature, the prospect is most enchanting. To the south and south-west as far as the eye can reach, nothing is to be seen but one extended landscape, interspersed with forest, fields, and farm houses, whilst at your feet sweeping in silent and unbroken majesty, roll the gushing waters of the Coosa. The agitation of these waters in their passage over the falls, causes the evolution of a large amount of vapor, which during the day, by the action of the sun's rays, is heated, attenuated, and suspended in the atmosphere; to be precipitated at night-fall in copious showers of dew, giving to the air of our vicinity an unusual, and at times unhealthy degree of dampness.

With these preliminary remarks I will proceed briefly to the enumeration of some of what we consider the most prominent causes, in the production of the disease under consideration; and first the geological situation of our city, it being in a low southern latitude, and surrounded by causes known to be favorable to the generation of malaria; secondly, the dampness of our atmosphere; and thirdly and lastly, diet and exposure. I do not wish, however to be understood as meaning to convey the idea that I consider the combined operation of all these causes necessary to the production of every case of diarrhœa as it prevails here; far from it; as we have had many proofs drawn from extensive observations of the reverse, any one of them in excess being sufficient to excite and continue a very troublesome form of the complaint.

Dr. Drake, in one of his traveling editorials,* speaking of the diseases of our section, remarks that a chronic diarrhœa which prevailed here much more than in any other part of the State, had been attributed by some of the medical gentlemen of Wetumpka, to a micaceous impregnation of the water. This opinion although apparently plausible, will, upon a moment's reflection, be discovered to be entirely untenable, from the fact that a large amount of this mica sparkles in the soil of all the adjoining counties, issues in the water of their springs, and is deposited upon standing from the same in great abundance; and yet those who are in its daily employment are comparatively as exempt from all forms of bowel disease, all other things being equal, as any other portion of the population of the State. True, in the Valley of the Alabama, and its larger tributaries, where the micaceous deposit is found perhaps in greater quantities than any where else, the population are more obnoxious to diarrhœa than where it is not so abundant. Still we think that the fact must and can be accounted for upon other principles, else we should have it prevailing at all seasons alike, or at least so long as the remote cause, this admixture of mica with our water, continued to operate. Taking it for granted then, that the above substance exercises no agency whatever in the production of diarrhœa, as it prevails here, we are irresistibly led to turn our attention to the study of the causes that are known to produce bowel disease in other climates, and see if we are not furnished with a solution of the difficulty.

The position laid down, and so ably maintained, by Dr. Cooke in his lectures; to wit, "that the same remote causes

---

* See Western Journal of Medicine and Surgery, July No., 1843.

produce both fever and fluxes," does in our humble opinion afford most ample and conclusive reasons, not only why the citizens of the Valley of the Alabama and its larger tributaries, but those of all other low malarious districts should be more liable to diarrhœa than those of the more high and healthy latitudes. Then if we have a cause operating throughout the spring, summer, and fall months, adequate to the production of diarrhœa in other latitudes, need we be surprised at its prevalence in this section of country, where all the elements necessary to the formation and evolution of malaria are so abundant, as they are throughout the whole southern portion of our State.

This remote cause acting upon the system through the medium of the lungs, and occasionally developing the disease, needs only some imprudence and exposure, or improper diet, conjoined with the known and acknowledged dampness of our atmosphere, to develop the worst and most intractable forms of diarrhœa.

*Symptoms.*—Dysentery and diarrhœa, two of the forms of disease to which the alimentary canal is liable, differ from each other more in degree than anything else, chronic dysentery being scarcely distinguishable from diarrhœa, and frequently here in their conversations by the faculty confounded; the distinction, however, is not of much if any practical importance.

In the incipient stages of diarrhœa the tongue is more or less furred; the pulse accelerated; the bowels excitable; the alvine evacuations usually preceded by a murmuring noise, and discharged with more or less griping and pain; the liver ceases to perform its proper functions, its healthy secretions being entirely suspended, no admixture of bile whatever appearing in the stools, which are now entirely protean in size, consistence, and color; as the disease advances the stomach usually becomes affected with sickness; the countenance grows pale, or sallow, and the skin generally dry and rigid; ultimately from the absorbents failing to take up the chyle as in health, great debility and emaciation, with dropsy of the lower extremities supervene.

At this stage of the complaint, you will frequently be informed by your patient that his tongue is sore, and importuned to do something for his mouth. Upon examination you will discover that his tongue presents a shrivelled and cracked appearance, resembling more a piece of raw beef than anything else; the fauces present a similar appearance, with the exception of now and then an occasional little ulcer; great thirst, with a general soreness extending down the œsophagus, and throughout the whole chest, and severe lancinating pains in the different portions of the alimentary canal are also present.

These symptoms, if not arrested speedily, terminate in death, in consequence of disorganization of the mucous membrane of the intestines, from chronic ulcerative inflammation, constituting one of the most intractable forms of the disease; and in fact the only strictly unmanageable one that has prevailed in this vicinty.

*Treatment.*—In the consideration of this, the third and last proposition contained in the heading of this article, I shall not attempt a rehearsal of the various plans of treatment and remedies that have been recommended and tried, by different members of the profession, but confine myself to such only as are of acknowledged utility, and in this shall be as brief and concise as the nature and importance of the subject will admit. The successful treatment of any form of disease depends, to a greater or less extent, upon a correct understanding of the causes that have conspired in its production, and in none more so than diarrhœa; and as the liver and skin, in our opinion, are always greatly if not wholly at fault in the one under consideration, to these our remedies should be addressed, and they should be of a nature that are known under ordinary circumstances but seldom to fail in the restoration of healthy action to these organs, and for this purpose we know of nothing better than some one of the mercurial preparations in combination with ipecac. and opium. Then if the patient be but recently attacked, tolerably plethoric, with furred tongue and febrile excitement, I would commence the cure with purgative doses of calomel, or calomel and Dover's powder at intervals, to be carried off at the proper time, if necessary, with castor oil or rhubarb. When the liver is sufficiently excited, the impression may be kept up by the exhibition, every two or three hours, of one of the following pills:

℞ Calomel, 20 grs.
Opium opt. 5 grs.
Ipecac. pulv. 20 grs.
Camph. gum, 20 grs.
Divide into 20 pills.

The effect of this combination is almost universally to arrest the inordinate peristaltic action of the intestines, relieve the griping, and soften the skin; should they succeed in keeping up the impression produced upon the liver, which they most generally will, it should be continued by the daily administration of four or five of them until relief is afforded, or a slight soreness of the glands of the mouth and throat is felt. During the administration of this remedy, the diet should be of as mild, nutritious and unstimulating a character as possible, such as water gruel, chicken water, &c. Flannel should be worn next to the skin, and as far as practicable all exposure to a cool or damp atmosphere be avoided.

The plan, now becoming pretty generally fashionable throughout the south, of having a fire kindled in the parlor or bed-chamber about sun down, will be found an admirable regulator for those laboring under diarrhœa. The taking also of a hot and strong decoction of ginger tea, upon retiring to bed, will be found serviceable in warding off those small local determinations that frequently create, during the early part of the night, a desire to evacuate the bowels.

During the summer and fall of 1838, whilst stationed with the 3rd regiment of artillery, United States army, at camp near Missionary Hill, Cherokee nation, east, I had an opportunity of witnessing, under the direction of the distinguished and lamented Dr. Samuel Forry, the good effects of the above plan of treatment. The doctor had spent several of the preceding years with the army in Florida, and had, from his great scientific attainments and practical skill, been placed in situations where he could enjoy the greatest field for observation. He informed me that the foregoing plan had been pursued by himself whilst at Tampa Bay, Black Creek, and Fort Jupiter, with great and unparalleled success, and that many of the soldiery had thereby been rescued from untimely graves, and returned to their families and homes in the enjoyment of comparatively good health.

In my hands both the mineral and vegetable astringents, such as the sacch. saturni, kino, catechu, alum and tannin, have all failed of their vaunted good qualities; neither have I seen the good effects result from the single or combined exhibition of the muriatic or nitric acids, as I had been taught to anticipate. In cases of great debility I have seen some little benefit derived from their tonic properties, internally administered, but nothing further, and they are not near so powerful in this point of view as the ferruginous preparations, and of these the carbonate of iron, or the muriated tincture are the best, and with which the system should be gradually charged, as we decline our mercurial preparations.

Mineral waters.—Of these those containing the largest proportion of iron, with a small trace of sulphur, are best adapted to produce the ends desired; and I would most earnestly advise all those laboring under chronic diarrhœa, to an early pilgrimage to one of those fashionable and healthy fountains of resort. The mere traveling through the mountainous regions of Tennessee and Kentucky, where these waters abound, superadded to the change of climate, and from the soft free-stone, to the hard lime-stone waters of those regions, has been known to effect most remarkable cures.

In conclusion, I shall feel highly gratified, and as if I had not lived in vain, should the preceding imperfect remarks answer no other valuable purpose than to cause some one more able to do the subject justice, to furnish the profession with a more extended paper.

Wetumpka, Alabama, July, 1845.

